# Finisher lamb growth and rumen fermentation responses to the plane of nutrition and naturally occurring coccidiosis

**DOI:** 10.3389/fvets.2023.1184557

**Published:** 2023-04-18

**Authors:** Sathya Sujani, Barbara R. dos Reis, Mark D. Ellett, Holly H. Schramm, Emma T. Helm, Robin R. White

**Affiliations:** ^1^School of Animal Sciences, Virginia Tech, Blacksburg, VA, United States; ^2^Virginia-Maryland College of Veterinary Medicine, Virginia Tech, Blacksburg, VA, United States

**Keywords:** average daily gain, *Eimeria* spp., FAMACHA© score, volatile fatty acid, anemia

## Abstract

The objective of the present study was to investigate the interaction of plane of nutrition and naturally occurring coccidiosis on finisher lamb growth performance, FAMACHA score, and rumen volatile fatty acid profile. The study included 30 Suffolk, Dorset or Suffolk x Dorset lambs and were divided into 2 groups based on their initial body weight and assigned to 2 feeding groups differing in dietary energy intake to create lambs representing divergent growth curves due to differing nutritional management. Lambs with naturally occurring coccidiosis and healthy lambs were present in both feeding groups making a 2 × 2 factorial arrangement of treatments, (a) high plane of nutrition (HPN) lambs with no clinical coccidiosis diagnosis (HPNH), (b) HPN lambs with clinical coccidiosis (HPNC), (c) low plane of nutrition (LPN) lambs with no clinical coccidiosis diagnosis (LPNH), (d) LPN lambs with clinical coccidiosis (LPNC). Body weight and FAMACHA scores were recorded once every 2 weeks. On d 65 of feeding, lambs were slaughtered, and rumen fluid samples were collected and analyzed for volatile fatty acid concentrations. All response variables were analyzed statistically using a linear mixed effects model with fixed effects for plane of nutrition, health status, and a random effect for initial body weight nested within the pen. The total and average weight gain were not associated with planes of nutrition, health status, or the interaction. Health status had an impact on FAMACHA© score (*P* = 0.047) and concentration of isobutyrate (*P* = 0.037) and tended to affect total VFA (*P* = 0.085) and acetate (*P* = 0.071) concentrations. The interaction between the plane of nutrition and the health status tended to affect butyrate concentration (*P* = 0.058). These data support the conclusion that coccidiosis infection impacted on rumen fermentation in a manner independent of the plane of nutrition; however, the translation of these rumen level impacts did not translate to the production responses.

## Introduction

Per capita lamb consumption in the U.S. is at a 20-year high, reaching 0.62 kg in 2021 (1). Despite high consumption, the U.S. sheep inventory is at all-time low of ~ 5.2 million sheep and lambs in 2019 (2). This discrepancy is largely because the domestic sheep industry fulfills only 50% of the U.S. demand for lamb meat and <30% of the demand for wool (3). Failure to meet this demand has resulted in increased levels of sheep meat and wool importation in the U.S. (1). Based on these market shifts, and the fact that the sheep industry is vital to the livelihood of many farmers throughout the country (4), there is potential for expanding the commercial sheep industry in the U.S. Additionally, sheep are used as a natural, low-cost means of managing rangelands, forests, and agricultural lands, because most of the nutrient requirements of mature sheep can be achieved through grazing, making sheep production suitable for low-input production environments.

Meeting adequate nutrition requirements of young lambs with high potential for live weight gain (4), however, requires more intensive feeding strategies. According to the NRC (4), young lambs, between 60 and 150 days of age, need diets with high protein and energy content which exceed the levels supplied in most forages used for sheep rearing. With seasonal fluctuations of forage quality most of the hay used for sheep feeding is low in crude protein and essential macro and micro minerals (5, 6). Therefore, to obtain high growth performance, diets with a high proportion of concentrate are typically used for finishing lambs. A great deal of research shows that lambs grow faster on concentrate-based diets than on forage-based diets (7–9). However, these feeding strategies often involve confinement feeding of lambs, resulting in challenges associated with maintaining flock health (2).

Maintaining a healthy flock is imperative for successful sheep operations because disease compromises overall growth rate, immunity, and reproductive performance, all leading to substantial economic losses (10, 11). The Animal and Plant Health Inspection Service of U.S. Department of Agriculture reported that internal parasites caused ~ 16 % loss in lambs (2) and a survey conducted by the same authority reported that gastrointestinal (GI) parasites are the number one health concern among sheep stakeholders (2). A 7-year review of clinical cases at Auburn University Veterinary Medical Teaching Hospital in Alabama found that parasite infection was the primary reason that 70% of sheep and 91% of goats were examined and treated by hospital clinicians (12). Gastrointestinal infestation with parasites is a particularly challenging situation because the infestation both increases cost of energy requirements due to activation of immune responses, and also depresses rumen function and negative consequences on metabolism (13) leading to lower energy supply to the animal (14). Coccidiosis is a major parasitic infection caused by *Eimeria* spp. protozoan that commonly infects the small and large intestines of sheep (15). Symptoms of clinical coccidiosis are diarrhea, dehydration, decreased appetite, weight loss, and death (16, 17). The effects of *Eimeria* infections on animals are thought to depend on the environmental situation, immunity of the animals (18), and the plane of nutrition. Studies focused on controlling coccidiosis are abundant in literature (19–22) but these studies are limited in exploring the interaction between plane of nutrition and naturally occurring coccidia on growth performance and rumen fermentation. Most of the coccidia associated studies have focused on the lower GI tract since it is the primary site of pathological change. Nevertheless, the impairment in the gastrointestinal tract may affect the function and activity of the rumen, contributing to impaired volatile fatty acid (VFA) production and reduced energy supplies to the animal. Because VFA are the main energy source for ruminants (23) and considering the cost of energy to maintain immunity and nutrient metabolism when lambs are under infection, it is worthwhile to investigate the effect of plane of nutrition and coccidiosis on rumen VFA production. Thus, the objective of this study was to explore how dietary manipulation and naturally occurring disease interact to influence animal performance and fermentation outcomes which will help to implement better health and nutritional management protocols.

## Materials and methods

### Animals, diets and experimental design

All the procedures and animal use described in this study were approved by the Virginia Tech Institutional Animal Care and Use Committee (Protocol #20-175). The study included 30 commercial wethers (Suffolk, Dorset or Suffolk x Dorset) that were group fed in a standard production feedlot (330 m^2^) at the Smithfield Farm, Virginia Tech, Blacksburg, VA. Prior to initiating the experiment all lambs were dewormed using fenbendazole (Panacur 10 mg/kg body weight, Merck Animal Heath, DE, USA) and Levamisole Hydrochloride (Prohibit at 8 mg/kg of body weight, AgriLabs, MO, USA), per standard industry management and veterinary recommendation. Deworming occurred 3 weeks prior to the start of the experiment and was not expected to interfere with the experimental measurements. Lambs were between 04 and 06 months of age at the start of the study. The lambs were classified by initial body weight targeting different finishing weights to cater market demands: low (28.4 ± 4.31 kg) and high (36.1 ±2.37 kg). Lambs were fed either (1) a low plane of nutrition (LPN) diet targeting 100 g/d weight gain or, (2) a high plane of nutrition (HPN) diet targeting 200 g/d weight gain. Due to market preferences, there is considerable variation in production systems regarding rates of lamb growth in the U.S (24). Including two planes of nutrition in this study allowed for generalization of results among different growth trajectories of finishing lambs. Grouping animals based on starting weights was reflective of industry settings where animals are grouped and purchased based on body weight. Body weight at the start of the study would be an emergent property of health, nutritional, genetic, and environmental factors, and is a complex phenotype that should not be conflated to reflect only differences in planes of nutrition.

Across plane of nutrition groups, lambs averaged 32.1 ± 4.5 kg body weight at the beginning of the experiment. After introduction to the feedlot environment, animals were naturally infected with *Eimeria* spp. Animals began showing signs of coccidiosis after 13 days, and both groups of animals received herd-level coccidiosis treatment with amprolium (Corid 9.6 % solution, 8 mg of amprolium per 1 kg of body weight for 5 consecutive days). Animals did not have a history of anticoccidial treatment prior to entering the feedlot.

Out of 30 lambs, 04 lambs died (1 due to coccidiosis, 2 due to pneumonia and coccidiosis, and 1 for undiagnosed reasons) during the experimental period and those lambs were excluded from the data set. From the 26 lambs that completed the trial, 9 lambs were diagnosed with clinical coccidiosis and recovered after treatment (infected lambs were individually treated following the same protocol as herd level treatment). The low nutritional group consisted of 14 lambs of which 6 lambs were diagnosed with coccidiosis. The HPN group consisted of 12 lambs, of which 3 lambs were diagnosed with coccidiosis. The resulting 4 treatment groups were (a) HPN lambs with no clinical coccidiosis diagnosis (HPNH), (b) HPN lambs with clinical coccidiosis (HPNC), (c) LPN lambs with no clinical coccidiosis diagnosis (LPNH), and (d) LPN lambs with clinical coccidiosis (LPNC). Diets in both groups were consisted with *ad libitum* grass hay and a commercial concentrate (Cargill Animal Nutrition, Minneapolis, MN, USA) supplement and were fed twice daily at 0900 h and 1700 h. Lambs in the LPN group received 0.45 kg of concentrate per lamb and 0.90 kg of concentrate per lamb in the HPN group. Target was to provide two levels of energy to achieve two different ADG. Both groups had access to fresh, clean water and mineral supplements throughout the day.

### Measurements, sampling and laboratory analysis

Body weight and FAMACHA© scores were recorded once every 2 weeks. The FAMACHA system was used to determine the lower eye mucous membrane color that correspond to different levels of anemia: 1 = red, non-anemic; 2 = red-pink, non-anemic; 3 = pink, mildly anemic; 4 = pink-white, anemic; 5 = white, severely anemic (25). Although the FAMACHA© is developed to assess anemia caused by *Haemonchus* species, the tool has been used as an anemia indicator caused by a wide range of gastrointestinal parasites including *Eimeria* species (26, 27). Previous studies by Nurzaty Ewani et al., (26) and Wang et al., (28) observed anemia and diarrhea associated with lambs infected with *Eimeria* spp. Further, if lambs have more *Haemonchus*, this likely suppresses immune system and lambs will more likely be clinical for coccidia. Lambs were slaughtered on d 65 of the experiment and rumen fluid samples were collected from each animal promptly after slaughter. Samples were collected and processed in the laboratory and stored in glass vials at −20°C until further analysis. Approximately 100 g of concentrate and grass hay were collected weekly, composited, and stored in −20°C until analysis.

Volatile fatty acid concentrations were analyzed using gas chromatography. Concentration of total VFA and individual VFA per lamb were determined using a Hewlett-Packard 5,890 series gas chromatograph (Agilent Technologies, Santa Clara, CA) equipped with a glass packed column (23110-U, Sigma-Aldrich) with N_2_ as a carrier gas (24 mL/min). The inlet was 150°C, the flame ionization detector was 180°C, and the oven temperature was 175°C. Each run was 18 min long to separate acetate (retention time: 1.6 min), propionate (3.0 min), isobutyrate (5.2 min), butyrate (6.8 min), pivalic acid (internal standard; 7.8 min), 2-methylbutyrate (11 min), isovalerate (12.9 min), and valerate (16.1 min). To prevent carryover effects between samples and to maintain similar conditions, between each sample distilled H_2_O was injected. Feed samples were dried for 24 h at 55°C in a forced-air oven (Thermo Scientific Heratherm Advanced Protocol Ovens Model 51028115, Fisher Scientific, Waltham, MA) and ground to pass through a 1 mm screen of a Wiley mill (Model 4, Thomas Scientific, Swedesboro, NJ). Proximate analysis of feed samples was done by an external laboratory (Cumberland Valley Analytical Services, Waynesboro, PA). Analyses included DM (29, 30), N (method 990.03; Leco FP-528 Nitrogen Combustion Analyzer, Leco Corp., St. Joseph, MI), ADF [method 973.18; (31)], NDF (32), lignin (29), starch (33), ash [method 942.05; (31)], and minerals by inductively coupled plasma [method 985.01; (31)].

### Statistical analysis

Statistical analyses were conducted in R version 4.1.2 (34) using the lme4 and lmerTest packages (35). Response variables included were total body weight gain, average daily gain, FAMACHA score and concentrations and molar proportions of individual VFA. The following model was fitted for all variables:


$$\begin{array}{l} \text{Yijklm}=\text{μ}+\text{Ni}+\text{Hj}+\left(\text{NHij}\right)+\text{BWk}\left(\text{l}\right)+\text{Pl}+\text{eijklm}, \end{array}$$

where Yij is the dependent variable, μ is the overall mean, Ni is the fixed effect of the plane of nutrition, Hj is the fixed effect of health status, NHij is the interaction effect, BWk is the random effect of initial body weight of individual lamb nested within the pen, and eijklm is the residual error. Analysis of variance (ANOVA) was performed for each variable and estimated marginal means were calculated using the emmeans package (36). Significance was declared at *P* < 0.05 and a tendency considered when 0.05 ≤ *P* < 0.10. Importantly, in our statistical analysis, we used the effect of initial body weight to explore how group responses might differ due to initial differences in the animals (expressed as differences in body weight) vs. those differences based on the nutritional treatments. Due to a failure in sampling of rumen fluid, data from 2 animals from HPNH group were not used for the statistical analysis.

## Results

### Growth performance and FAMACHA score

The effects of the plane of nutrition and health status on growth performance and FAMACHA© score of lambs are presented in Table 1. The plane of nutrition, status of health or the interaction did not have an effect on total gain or ADG. Numerically, the highest WG and ADG were associated with HPNH group, and values in other groups were not significantly different from each other. Although the numerical differences support the idea that nutrition and health interact to support performance, the variability around individual animal performance within the study precluded identification of statistically significant relationships. Status of health had an effect on FAMACHA© scores (*P* = 0.047) and lower values were associated with healthy groups irrespective of the plane of nutrition, with numerically higher FAMACHA© scores were associated with coccidiosis conditions.

**Table 1:** Weight gain and FAMACHA score of lambs in response to the plane of nutrition and health status.

**Item**	**HPNH**	**HPNC**	**LPNH**	**LPNC**	**SEM**	***P*** **-value**		
						**Nutrition**	**Health**	**Nutrition** × **Health**
Total WG, kg	20.3	15.90	16.7	16.8	3.83	0.738	0.482	0.463
ADG, g	87.5	68.8	72.0	72.4	16.52	0.738	0.482	0.463
FAMACHA	1.70	1.93	1.70	2.23	0.311	0.720	0.047^*^	0.348

HPNH, high plane of nutrition healthy; HPNC, high plane of nutrition coccidiosis infected; LPNH, low plane of nutrition healthy; LPNC, low plane of nutrition coccidiosis infected; WG, weight gain; ADG, average daily gain. Number of lambs included in each group was; HPNH = 7, HPNC = 3, LPNH = 8, LPNC = 6.

* *P* ≤ 0.05.

### Rumen volatile fatty acid profile

Concentrations and molar proportions of individual VFA are presented in Table 2. Health status caused by the coccidiosis infection had an effect on concentration of isobutyrate (*P* = 0.037) and tended to alter the concentrations of total VFA (*P* = 0.085) and acetate (*P* = 0.071). The interaction effect of the plane of nutrition and health status tended to affect butyrate concentrations (*P* = 0.058). Molar proportions of individual VFA were not affected by the plane of nutrition, health status or the interaction. Numerically, concentrations of total VFA and individual VFA in HPNH, HPNC and LPNH groups were quite similar to one another, while the LPNC group had the lowest reported values.

**Table 2:** Volatile fatty acid profiles of lambs in response to the plane of nutrition and health status.

**Item**	**HPNH**	**HPNC**	**LPNH**	**LPNC**	**SEM**	***P*** **-value**		
						**Nutrition**	**Health**	**Nutrition** × **Health**
Total VFA, mM	46.5	45.3	47.2	29.0	7.55	0.380	0.085	0.129
Acetate, mM	29.7	28.4	29.4	18.6	5.08	0.418	0.071	0.150
Propionate, mM	11.63	11.46	12.31	7.31	2.02	0.426	0.121	0.147
Butyrate, mM	2.88	3.57	3.18	1.73	0.675	0.357	0.433	0.058
Valerate, mM	0.704	0.760	0.696	0.532	0.144	0.436	0.644	0.354
Isovalerate, mM	0.482	0.397	0.576	0.286	0.131	0.950	0.120	0.376
Isobutyrate, mM	0.799	0.513	0.897	0.592	0.157	0.573	0.037^*^	0.938
A/P	2.50	2.52	2.50	2.59	0.182	0.484	0.744	0.324
Acetate, %	64.3	64.0	62.5	62.5	2.22	0.516	0.938	0.928
Propionate, %	25.0	25.4	25.9	25.0	1.40	1.000	0.843	0.586
Butyrate, %	5.97	6.69	6.35	6.61	0.759	0.861	0.436	0.710
Valerate, %	1.45	1.73	1.50	1.80	0.397	0.773	0.120	0.959
Isovalerate, %	0.983	0.892	1.2	1.15	0.393	0.645	0.749	0.929
Isobutyrate, %	1.67	1.17	1.84	2.12	0.701	0.558	0.697	0.196

HPNH, high plane of nutrition healthy, HPNC, high plane of nutrition coccidiosis infected, LPNH, low plane of nutrition healthy, LPNC, low plane of nutrition coccidiosis infected, WG, weight gain, ADG, average daily gain. Number of lambs included in each group was; HPNH = 7, HPNC = 3, LPNH = 8, LPNC = 6.

* *P* ≤ 0.05.

## Discussion

### Growth performance and FAMACHA© score

According to the NRC (4) guidelines for sheep nutrient requirements, 4–7 month old finisher lambs should achieve an ADG of range of 205 to 295 g. In the present study, irrespective of the plane of nutrition and health status neither group reached the recommended ADG, partially because diets were formulated to target lower rates of gain than suggested in the NRC (4). Despite the highest ADG of 87.5 g associated with the HPNH group; it is still <50% of the target used in formulation. Possible explanations for the limited growth rates observed in the present study could be poor feed intake and forage quality. Based on the chemical analysis of the forage and concentrates (Table 3) fed to the lambs, we observed that grass hay was lower in protein supply of 7.7% and higher NDF of 69%. This forage was quite mature and likely had poor digestibility. Another possible explanation for lower than the standard ADG we observed in the HPNH group is the negative impact of whole herd coccidiosis treatment and subclinical coccidiosis. Coccidiosis treatment is known to depress feed and water intake, thus may have negatively impacted productivity of animals. Further, the effect of coccidiosis on weight gain and feed efficiency has been inconsistent in studies and it is even more challenging to ascertain the influence of subclinical infections (37). Nevertheless, subclinical coccidiosis may contribute to low weight gain, reduced feed intake and feed utilization (38, 39). However, the lower ADG observed in the present study closely follows the ADG values reported in several previous studies (40, 41). In Atti and Mahouachi (40) they observed the highest ADG of 108 g/day for the high nutrition group and 61 g/day for the low nutrition group while Bhatt and Sahoo (41) reported 99 to 140 g/day ADG values. In contrast to present results which did not show an effect of nutritional plane on total weight gain, numerous previous studies have linked plane of nutrition and growth performance. For example, a previous study (42) reported that the plane of nutrition had a dramatic effect on lamb live weight, with low and high lambs differing in weight by 9.1 kg (*P* < 0.001) at weaning and by 14.9 kg at slaughter. Indeed, standard understanding of energetics also supports the expectation that lambs fed greater energy content diets should grow more rapidly. Although we observed numerically higher total weight gains and ADG in the HPN group compared with the LPN group, the variability induced by the added health challenge, and the small number of animals used in each group may help explain why these numerical differences did not approach statistical significance. Indeed, our numerical differences are sensible given the basic understanding of nutritional energetics and concur well with other previous work (43, 44) reporting positive gain responses in feedlot lambs with dietary concentrate.

**Table 3:** Chemical composition of grass hay and commercial concentrate fed to lambs during the experiment.

**Item**	**Grass hay**	Commercial concentrate^*^
Dry matter, %	93.9	91.6
**Composition, % of DM**		
Crude protein	7.70	15.7
NDF	69.0	12.3
ADF	39.1	6.70
Lignin	4.80	0.92
Starch	3.10	49.5
Ash	5.74	8.03
Calcium	0.39	1.09
Phosphorus	0.32	0.52
Magnesium	0.27	0.36
Potassium	1.83	0.85

* Ingredients of the commercial concentrate: Processes grain by-products, grain products, roughage products, plant protein products, cane molasses (with propionic acid, sodium benzoate, potassium sorbate as preservatives), calcium carbonate salt, ammonium chloride, lignin sulfonate, soybean oil, sodium selenite, potassium sulfate, vitamin E supplement, magnesium sulfate, vitamin A supplement, vitamin D3 supplement, zinc oxide, manganous oxide, ferrous sulfate, calcium iodate, cobalt carbonate.

Lambs in 2 nutritional planes (HPN and LPN) received the diet in this table consisted of grass hay (*ad libitum*) and different levels of commercial concentrate (0.90 kg/animal/day for HPN group and 0.45 kg/animal/day for LPN group).

A major limitation of implementing a selective treatment approach for parasitic infections has been the lack of an efficient and economical means of identifying those animals' requiring treatment. To address this issue, FAMACHA score has been utilized successfully in African countries (45, 46) and the United States (47, 48). As shown in Table 1, FAMACHA© score did not differ between groups in response to the plane of nutrition but was affected by the health status (*P* = 0.047). The highest FAMACHA© score was observed in the LPNC group and is indicative of anemia due to the impaired ability of host to absorb nutrients caused by coccidiosis infection. In agreement with our results, the severity of coccidiosis in previous studies showed a significant correlation (*r* = 0.48, *P* < 0.01) with FAMACHA© score (26, 27). In these studies, they observed FAMACHA© score ranged between 2 and 3 which is slightly higher than the observed FAMACHA scores in the present study, which ranged between 1.43 (HPN, healthy) to 2.43 (LPN, infected). Another rational explanation for the anemic conditions we observed in infected groups may have caused by the occurrence of *H. contortus* even though it was not determined in the present study. *H. contortus* is the most pathogenic blood-sucking gastrointestinal nematode in ruminants and it causes anemic condition and also contributes to the severity of coccidia. This explanation is supported by a study where they reported anemic conditions in lambs and goat kids infected with both *Eimeria* spp. and *H. contortus* (49).

### Rumen VFA

Rumen VFA profile is known to be altered in response to the different ratios of forage to concentrate due to the changes in nutrient supply. However, other than isobutyrate, no differences of VFA concentrations or molar proportions in response to the plane of nutrition and health status were observed in this study. This is not particularly surprising because animals were fed similar diets, differing only by the mass of concentrate allocated daily. The lack of dietary influence on VFA might reflect the ability of the rumen and the animal to adapt to appropriate dietary concentrate: forage ratios through the self-adjustment of forage intake under *ad libitum* access to forage. In agreement with our results, previous studies reported no changes in total VFA of Tibetian sheep fed with different ratios of forage and concentrate (50). In ruminants, total VFA concentrations may be as low as 30 mM or be in excess of 200 mM but is typically between 70 and 130 mM (51). In our data we observed total VFA concentrations across groups were closer to the lower end. These data suggest that altering the plane of nutrition, within the bounds of this study, while maintaining *ad libitum* access to hay, supported fairly consistent rumen VFA conditions. Importantly, this should not be conflated with similar energy supplied by VFA from the different planes of nutrition, because rumen VFA concentrations do not take into account production, absorption, and interconversion of VFA, and are considerably influenced by rumen fluid pool size and dynamics. As such, these data are best interpreted to support a consistent form of fermentation among the two planes of nutrition.

Effect of coccidiosis on rumen fermentation and VFA profile is not extensively studied mainly because the negative impacts localized in the small and large intestines (16, 52). Coccidiosis from the *Eimeria spp*., results in destruction of the epithelial cells of the intestine hence the coccidia infection strongly interacts with the digestive microflora. A previous study reported a significant change in digestive microflora in goat kids where they observed progressive reduction of the Gram-positive population from 84% pre-infection to 24.3% after the onset of diarrhea. On the other hand, the Gram-negative population was conversely increased from 16% pre-infection to 75.7% after diarrhea (53). Therefore, we can assume that infection of coccidiosis also has the capacity to alter rumen microflora of lambs. This mechanism supports the lower concentrations of total VFA and individual VFA associated with coccidiosis infected groups. Lower concentrations may also reflect dysregulated fluid dynamics, which are consistent with GI infection.

We did not observe differences in molar proportions of individual VFA across groups, but the low butyrate molar proportions in all groups is noteworthy. Moreover, the interaction effect of nutrition and coccidiosis infection had a tendency toward altering butyrate concentration (*P* = 0.058) resulting lowest concentration in LPNC group, and both observations suggest modifications to butyrate in the rumen. Alterations in butyrate production make sense given its role in functional development of rumen epithelium (54, 55) and also in lower GI tract (56, 57). Studies have reported that increased concentration of butyrate is highly correlated with the enlargement of the ruminal epithelium absorptive surface area (58). We can speculate that the interactive effect of health and nutrition across all the groups suppressed the activity of certain rumen microorganisms which are responsible for producing butyrate hence low concentration and molar proportion of butyrate. It is possible that these shifts may also confer under-development and reduced functionality of the rumen and intestinal epithelium cells. This hurdle of poor rumen functionality possibly lowered feed utilization by lambs leading to the low weight gains observed. One can argue that the above concept can be reverse engineered; impaired health directly suppressed the development and functionality of the rumen epithelium and limited the utilization of butyrate by the cell wall hence accumulating more butyrate in the rumen. The observed numerically higher butyrate molar proportions in coccidiosis infected groups support the possible importance of this feedback in driving animal physiology. The same explanation of acid accumulation and hence slowing down the synthesis of VFA can be applied to the other VFA concentration data as well (59). Nevertheless, the lack of confirming measurements and prevailing lack of statistically significant differences among molar proportions precludes definitive discussion on these linkages. Future work should further explore the role of butyrate dynamics and epithelial function during GI parasite infection to more thoroughly explore these concepts.

## Conclusion

This study sought to investigate the effect of plane of nutrition and coccidiosis infection on finisher lamb growth performance, FAMACHA score, and rumen volatile fatty acid profile. Plane of nutrition did not have an impact on any of the response variables considered. Health status showed a significant effect on FAMACHA score and concentrations of isobutyrate and tendency toward total VFA, acetate, and butyrate suggesting both or either, alteration in rumen microbial profile or rumen epithelium as explanations. An interaction between the plane of nutrition and health status was identified for butyrate concentrations. Overall, this result leads us to assume that changes in nutritional plane could have an impact on growth performance but in our study the effect of health status were more prominent suggesting that it is challenging to overcome the effect of suppressed immunity through nutritional strategies.

In future studies, it would be beneficial to focus on the effect of coccidiosis infection during rumen and overall gut development (preferably lambs below 3 months old age) and consequently how it will impact overall rumen function later in life. This will be helpful to develop clinical and nutritional interventions to support animals that have been exposed to coccidiosis and other related infections.

## Data availability statement

The raw data supporting the conclusions of this article will be made available by the authors, without undue reservation.

## Ethics statement

The animal study was reviewed and approved by Virginia Tech Institutional Animal Care and Use Committee (Protocol #20-175).

## Author contributions

SS and RW: conceptualization and design of the study. SS and BR: performed the experiment. SS, BR, ME, and EH: collection of samples. SS: sample preparation, formal analysis, manuscript writing, and editing. RW: statistical analysis and editing the manuscript. HS: animal care and animal health consulting. All authors contributed to the editing of the final version of the manuscript.
